# Correlation between Chest Computed Tomography Scan Findings and Mortality of COVID-19 Cases; a Cross sectional Study

**Published:** 2020-05-14

**Authors:** Masoomeh Raoufi, Seyed Amir Ahmad Safavi Naini, Zahra Azizan, Fatemeh Jafar Zade, Fatemeh Shojaeian, Masoud Ghanbari Boroujeni, Farzaneh Robatjazi, Mehrdad Haghighi, Ali Arhami Dolatabadi, Hossein Soleimantabar, Simindokht Shoaee, Hamidreza Hatamabadi

**Affiliations:** 1 Department of Radiology, School of Medicine, Imam Hossein Hospital, Shahid Beheshti University of Medical Sciences, Tehran, Iran.; 2 Imam Hossein Clinical Research Development Center, Imam Hossein Hospital, Shahid Beheshti university of Medical Science, Tehran, Iran.; 3 Department of Radiology, School of Medicine, Imam Hossein Hospital, Shahid Beheshti University of Medical Sciences, Tehran, Iran.; 4 Department of Infectious Diseases, Imam Hossein Teaching and Medical Hospital, Shahid Beheshti University of Medical Sciences, Tehran, Iran.; 5 Department of Emergency Medicine, Imam Hossein Hospital, Shahid Beheshti University of Medical Sciences, Tehran, Iran.

**Keywords:** Tomography scanners, x-ray computed, epidemiology, COVID-19, severe acute respiratory syndrome coronavirus 2, mortality, prognosis, patient outcome assessment

## Abstract

**Introduction:**

Predicting the outcomes of COVID-19 cases using different clinical, laboratory, and imaging parameters is one of the most interesting fields of research in this regard. This study aimed to evaluate the correlation between chest computed tomography (CT) scan findings and outcomes of COVID-19 cases.

**Methods:**

This cross sectional study was carried out on confirmed COVID-19 cases with clinical manifestations and chest CT scan findings based on Iran's National Guidelines for defining COVID-19. Baseline and chest CT scan characteristics of patients were investigated and their correlation with mortality was analyzed and reported using SPSS 21.0.

**Results:**

380 patients with the mean age of 53.62 ± 16.66 years were evaluated (66.1% male). The most frequent chest CT scan abnormalities were in peripheral (86.6%) and peribronchovascular interstitium (34.6%), with ground glass pattern (54.1%), and round (53.6%) or linear (46.7%) shape. There was a significant correlation between shape of abnormalities (p = 0.003), CT scan Severity Score (CTSS) (p <0.0001), and pulmonary artery CT diameter (p = 0. 01) with mortality. The mean CTSS of non-survived cases was significantly higher (13.68 ± 4.59 versus 8.72 ± 4.42; <0.0001). The area under the receiver operating characteristic (ROC) curve of CTSS in predicting the patients’ mortality was 0.800 (95% CI: 0.716-0.884). The best cut off point of chest CTSS in this regard was 12 with 75.82% (95% CI: 56.07%-88.98%) sensitivity and 75.78% (95% CI: 70.88%-80.10%) specificity. The mean main pulmonary artery diameter in patients with CTSS ≥ 12 was higher than cases with CTSS < 12 (27.89 ± 3.73 vs 26.24 ± 3.14 mm; p < 0.0001).

**Conclusion:**

Based on the results of the present study it seems that there is a significant correlation between chest CT scan characteristics and mortality of COVID-19 cases. Patients with lower CTSS, lower pulmonary artery CT diameter, and round shape opacity had lower mortality.

## Introduction

Corona Virus Disease 2019 (COVID-19), was declared to be a global health emergency by the World Health Organization (WHO) on January 30^th^, 2020. It is necessary to recognize predictors of poor prognosis based on clinical manifestations, laboratory tests, and radiologic patterns of lung involvement to properly deal with patients suspected to have COVID-19 (1).

Analyses have introduced comorbidities such as Chronic Obstructive Pulmonary Disease (COPD), diabetes, hypertension and malignancy, high Sequential Organ Failure Assessment (SOFA) score, and higher levels of Erythrocyte Sedimentation Rate (ESR), d-dimer, albumin and IL-6 as poor prognostic factors, especially in older males (2-5). 

Although RT-PCR has become a standard test for detecting patients, some studies have reported that clinical and radiological investigation could be used as an easier and more readily available way to detect patients, especially since it takes less time and has a lower cost compared to RT-PCR (6-9). With the passage of time from the onset of the symptoms, chest computed tomography (CT) scan findings become more frequent. The chest CT findings include consolidation, linear opacities, crazy-paving pattern, and the reverse halo with ground glass opacification being the predominant pattern (9-12). The chest CT scan Severity Score (CTSS) was suggested as a quick means to evaluate the severity of pulmonary involvement with an optimal threshold of 19.5 out of 40 (13).

CT scan features were indicated to be helpful in evaluation of the severity and extent of the disease (14) as well as monitoring the clinical course (15). Consolidation, linear opacities, crazy-paving pattern, and bronchial wall thickening were reported to be significantly higher in severe/critical patients, who also had higher CT scores and more extra-pulmonary lesions (16). It is worth noting that many prediction models were presented in the academic literature and the most frequently reported predictors of prognosis included CT scan features (17). Furthermore, pulmonary indication value was reported to be significantly correlated with the main clinical symptoms and laboratory results (18). 

Based on the above-mentioned facts, this study aimed to evaluate the correlation between chest CT scan findings and outcome of COVID-19 cases.

## Methods

***Study design and setting***

This retrospective single-center cross sectional study was carried out on COVID-19 patients diagnosed with clinical manifestations and chest CT scans based on Iran's National Guidelines. These patients were admitted to the emergency department (ED) of Imam Hossein Hospital, Tehran, Iran, from February 22^th^ 2020, until March 22^th^ 2020. The Ethics Committee of Shahid Beheshti University of Medical Science approved the study (Ethics ID: IR.SBMU.RETECH.REC.1399.003). Informed consent was obtained from all those who were enrolled and confidentiality of patients' data was maintained.

***Participants***

Cases with suspected COVID-19 based on Iran's national guidelines for defining COVID-19, whose chest CT scan findings were strongly in favor of COVID-19, were enrolled in the study. Based on this definition, patients with acute respiratory infection who do not positively respond to the usual pneumonia treatment or who have had recent travel history to China as well as patients having respiratory symptoms with any severity, who have had physical contact with an individual diagnosed with or suspected to have COVID-19 were considered suspected cases for COVID-19. Definite diagnosis of the patients was based on chest CT scan and RT-PCR (for admitted cases). Patients were excluded if they had a normal chest CT scan upon arrival to ED, two negative RT-PCRs, or declined to participate in the study.

***Data gathering***

Using a predesigned checklist, demographic data (age, gender), underlying disease (diabetes mellitus, cardiovascular disease, smoking, kidney disease, asthma, respiratory diseases other than asthma, malignancy, hematologic disorders, rheumatologic disorders, neurologic disorders, use of steroids, hypertension), symptoms (fever, dyspnea, myalgia, headache, nausea, vomiting, chest pain, and etc.), vital signs, laboratory findings and outcome were collected for all cases. In addition, the chest CT scan findings, which were reported by an expert radiologist who was completely blind to clinical and laboratory findings, were recorded for all cases.  

Low dose lung CT scans were performed for all patients using a 16 detector CT scanner (SIEMENS; Emotion; SOMATOM) with patients in a supine position; other CT parameters were kvp: 100; mAs: 50-100; pitch:1.5; thickness: 4mm. The window was set as mediastinal (window level, 50 HU; window width, 400 HU) and lung (window level, -400 to - 700 HU; window width, 1200±1500 HU). Patients were instructed to hold their breath in order to minimize motion artifacts.

An expert attending radiologist (with 10 years of experience), reviewed the chest CT scans of the patients for involvement and severity of each lobe, pattern of involvement (such as ground glass, consolidation, crazy paving and reverse halo), form of parenchymal involvement (such as round opacity, linear opacity and no specific form), distribution of lung abnormalities (peripheral, peribronchovascular and peri-hilar), associated findings (such as pleural effusion, pericardial effusion, mediastinal and hilar significant adenopathy and pulmonary solid nodules), severity of involvement (based on CTSS), and pulmonary artery diameter (including main pulmonary trunk (MPA), right and left pulmonary arteries (RPA and LPA)).

The widest short-axis diameter of the main, right and left pulmonary artery were measured on the transverse section at the level of bifurcation of pulmonary artery trunk. 

CTSS, a semi-quantitative scoring, was used to estimate the severity of lung parenchymal involvement. Each of the 5 lung lobes were visually scored from 0 to 5 as: 0) no involvement; 1) < 5% involvment, 2) 5-25% involvement, 3) 26-49% involvement 4) 50-75% involvement, and 5) >75% involvement. The total CTSS was the sum of the individual lobar scores and ranged from 0 (no involvement) to 25 (maximum involvement). This scoring system was acquired from Fenj Pan and Tianhe Ye study (19).

 Five medical students were responsible for data gathering under the direct supervision of an emergency medicine specialist. Medical information of the patients was collected from their electronic hospital records. 

**Statistical analysis**

Analyses were performed using SPSS 21.0. The findings were presented as mean ± standard deviation or frequency (%). Student t-test, chi-square, and Fisher’s exact test were used for comparisons. Significance level was considered as p <0.05. Receiver Operating Characteristic (ROC) curve was used for finding the best cut off point of total chest CTSS in predicting the patients with higher risk of mortality.

## Results

***Baseline characteristics of studied cases***

Three hundred eighty patients with the mean age of 53.62 ± 16.66 (18 – 97) years were evaluated (66.1% male). Diabetes mellitus (22.81%), cardiovascular disease (13.2%), and hypertension (12.1%) were among the most frequent comorbidities among the patients. The most frequent presenting clinical symptoms were cough (60.3%), fever (55.8%), and dyspnea (48.2%), respectively. 154 (53.8%) cases were admitted to the hospital and 133 (46.2%) patients were discharged and managed at home. The total and in-hospital mortality rates during the 2-week follow up were 7.6% (29 from 380 cases) and 14.2% (22 from 154 cases) in this series, respectively. Tables 1 and 2 compare the baseline characteristics and laboratory parameters between survived and non-survived cases. 

***Chest CT scan findings***

The most frequent chest CT scan abnormalities were in peripheral (86.6%) and peribronchovascular interstitium (34.6%), with ground glass pattern (54.1%), and round (53.6%) or linear (46.7%) shape. The time between onset of initial symptoms and performing chest CT scan was less than 4 days in 30.8% (early stage), 4 - 6 days in 35.8% (intermediate stage), and more than 6 days in 33.3% (late stage) of patients. Stage of disease had no correlation with pattern of chest CT scan involvement (p= 0.692). There was a significant correlation between stage of disease and CTSS (p= 0.008). Table 3 compares the chest CT scan characteristics of cases between survived and non-survived cases. There was a significant correlation between shape of abnormalities (p = 0.003), CTSS (p <0.0001), and pulmonary artery CT diameter (p = 0. 01) with mortality.

***Correlation of CTSS and mortality***

**Figure 1:**
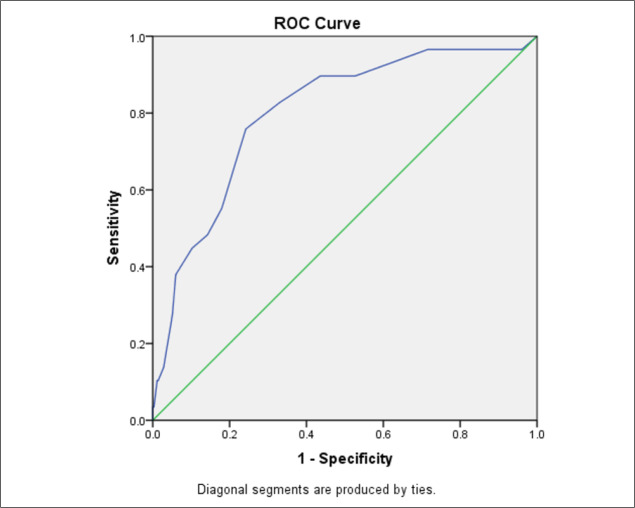
Area under the receiver operating characteristic (ROC) curve of chest computed tomography severity score in predicting COVID-19 mortality

The mean CTSS of non-survived cases was significantly higher (13.68 ± 4.59 versus 8.72 ± 4.42; <0.0001). The area under the ROC curve of CTSS in predicting the patients’ mortality was 0.800 (95% CI: 0.716-0.884; figure 1). The best cut off point of chest CTSS in this regard was 12 with 75.82% (95% CI: 56.07%-88.98%) sensitivity and 75.78% (95% CI: 70.88%-80.10%) specificity. 

***Correlation of pulmonary artery diameter and CTSS***

The mean main pulmonary artery diameter in patients with CTSS ≥ 12 was higher than cases with CTSS < 12 (27.89 ± 3.73 vs 26.24 ± 3.14 mm; p < 0.0001). The mean right pulmonary artery diameter was significantly higher in cases with higher CTSS of right middle (p= 0.045) and right lower (p < 0.0001) lobes of the lung. In addition, the mean left pulmonary artery diameter was significantly higher in cases with higher CTSS of left upper (p= 0.006) and left lower (p < 0.002) lobes of the lung. 

**Table 1:** Comparing the baseline characteristics of COVID-19 patients between survived and non-survived cases

**Variables**	**Total (n=380)**	**Survived (n=351)**	**Died (n=29)**	**P value**
**Gender **				
Female	129 (66.1)	122 (94.6)	7 (5.4)	0.246
Male	251 (33.9)	229 (91.2)	22 (8.8)	
**Age (year)**				
Mean ± SD	53.57 ± 16.66	52.85 ± 16.35	63.60 ± 17.68	0.002
**Comorbid disease**				
Yes	118 (40.8)	106 (89.8)	12 (10.2)	0.949
No	171 (59.2)	154 (90.1)	17 (9.9)	
**Presenting vital sign**				
Temperature (c)	37.34 ± 1.04	37.29 ± 1.00	37.94 ± 1.39	0.104
Systolic BP (mmHg)	118.21 ± 11.61	118.40 ± 11.6	115.00 ± 12.90	0.572
Respiratory rate (/min)	18.80 ± 4.32	18.36 ± 3.62	26.67 ± 8.62	0.001
Heart rate (/min)	89.99 ± 14.50	88.92 ± 13.30	103 ± 22.81	0.016
Saturation O2 (%)	93.43 ± 6.11	93.82 ± 5.88	87.13 ± 6.72	0.002
**Clinical manifestations**				
Fever	212 (55.8)	193 (72.0)	19 (65.5)	0.642
Cough	229 (60.3)	209 (77.1)	20 (74.1)	0.720
Dyspnea	183 (48.2)	163 (62.0)	20 (69.0)	0.460
Myalgia	182 (47.9)	168 (63.2)	14 (51.9)	0.249
Headache	128 (33.7)	121 (45.1)	7 (26.9)	0.074
Chest pain	77 (20.3)	68 (26.1)	9 (34.6)	0.347
Nausea or vomiting	98 (25.8)	88 (33.5)	10 (37.0)	0.708
**Disposition**				
Hospital admission	140 (48.6)	124 (47.0)	16 (66.7)	< 0.001
ICU admission	14 (5.2)	9 (3.4)	6 (25.0)	
Home admission	133 (46.2)	131 (49.6)	2 (8.3)	

Data are presented as mean ± standard deviation (SD) or frequency (%). BP: blood pressure; ICU: intensive care unit.

**Table 2:** Comparing the laboratory findings of COVID-19 patients between survived and non-survived cases

**Characteristics**	**Survived (n=351)**	**Died (n=29)**	**P value**
**Complete blood count**			
WBC (10^9^/L)	6.90 ± 3.96	11.46 ± 7.96	< 0.0001
Hemoglobin (g/dl)	13.14± 1.91	12.85 ± 2.09	0.285
Hematocrit (%)	39.03 ± 5.13	38.21 ± 5.49	0.401
Platelet (10^9^/L)	195.15 ± 83.13	183.50 ± 62.60	0.487
Lymphocyte (%)	22.66 ± 11.37	11.42 ± 5.59	< 0.0001
Neutrophil (%)	69.81 ± 12.76	82.58 ± 6.55	< 0.0001
**Blood gas analysis**			
pH	7.43 ± 0.09	7.38 ± 0.17	0.038
PO_2_ (mm Hg)	34.50 ± 18.57	36.33 ± 24.96	0.654
PCO_2_ (mm Hg)	41.99 ± 10.76	37.32. ± 11.75	0.053
HCO_3_ (mEq/L)	27.01. ± 7.10	23.38 ± 7.01	0.016
**Coagulation profile**			
PT (s)	12.73 ± 3.17	15.07 ± 3.82	0.010
PTT (s)	28.27 ± 9.91	29.82 ± 5.32	0.526
INR (IU)	2.33 ± 10.57	1.35 ± 0.38	0.696
**Liver enzymes**			
AST (U/L)	64.38 ± 234.43	104.56 ± 117.76	0.533
ALT (U/L)	58.09 ± 208.98	63.78 ± 47.53	0.926
**Kidney enzyme**			
Urea ( mg/dL)	42.78 ± 33.42	66.81 ± 53.83	0.001
Creatinine (mg/dL)	1.97 ± 9.32	1.69 ± 0.95	0.877
**Others**			
Sodium (mEq/L)	137.43 ± 4.00	136.26 ± 7.13	0.187
Potassium (mEq/L)	3.97 ± 0.61	4.09 ± 0.79	0.325
Calcium (mg/dL)	8.23 ± 1.20	7.90 ± 0.82	0.314
Magnesium (mg/dL)	2.20 ± 1.50	2.09 ± 0.42	0.774
ESR (mm/hr)	49.56 ± 26.30	54.21 ± 31.03	0.486
CRP (mg/L)	46.47 ± 42.77	89.89 ± 68.71	< 0.0001
CPK (U/L)	216.38 ± 474.51	1033.45 ± 1754.19	< 0.0001
Blood sugar(mg/dL)	154.48 ± 71.49	159.54 ± 85.21	0.816

Data are presented as mean ± standard deviation. WBC: white blood cell; PT: prothrombin time; PTT: partial thromboplastin time; INR: international normalized ratio; AST: aspartate aminotransferase; ALT: alanine aminotransferase; ESR: erythrocyte sedimentation rate; CRP: C-reactive protein; CPK: creatinine phosphokinase.

**Table 3:** Comparing the chest computed tomography (CT) scan findings of COVID-19 patients between survived and non-survived cases

**Characteristics**	**Survived (n=351)**	**Died (n=29)**	**P value**
**Location **			
Peripheral	304 (86.6)	25 (88.2)	1.000
Peribronchovascular	122 (34.8)	10 (34.5)	0.976
Perihilar	1 (0.3)	0 (0.0)	1.000
Upper zone	23 (6.6)	0 (0.0)	0.240
Lower zone	60 (17.1)	5 (17.2)	0.984
Upper and lower zone	87 (24.8)	8 (27.6)	0.738
**Presentation**			
Ground glass	190 (54.1)	15 (51.4)	0.996
Consolidation	104 (29.6)	9 (31.0)	
**Shape**			
Round opacity	188 (53.6)	6 (20.7)	0.003
Linear opacity	150 (42.7)	15 (51.7)	0.348
Non specified	20 (5.7)	5 (17.2)	0.016
Crazy paving	11 (3.1)	3 (10.3)	0.082
**Lobar CTSS**			
Right upper lobe	1.71 ± 0.78	2.25 ± 0.96	0.001
Right middle lobe	2.02 ± 0.82	2.71 ± 0.93	< 0.0001
Right lower lobe	2.45 ± 0.86	3.25 ± 0.79	< 0.0001
Left Upper lobe	2.15 ± 0.86	3.76 ± 1.14	0.001
Left lower lobe	2.31 ± 0.89	3.21 1.13	< 0.0001
**Total CTSS**			
Mean ± SD	8.72 ± 4.42	13.68 ± 4.59	<0.0001
**PA diameter (mm)**			
Main	26.54 ± 3.29	28.93 ± 3.99	<0.0001
Right	17.84 ± 3.23	19.61 ± 4.11	0.007
Left	17.61 ± 2.86	19.61 ± 2.58	<0.0001

Data are presented as mean ± standard deviation (SD) or frequency (%). CTSS: Computed tomography severity score. PA: pulmonary artery.

## Discussion

Based on the results of the present study, it seems that there is a significant correlation between chest CT scan characteristics and mortality of COVID-19 cases. Patients with lower CTSS, lower pulmonary artery CT diameter, and round shape opacity had lower mortality. 

Ground Glass Opacity (GGO) and consolidation were the most frequent chest CT scan findings, which was consistent with other studies (20, 21). The most common location of abnormalities was peripheral, followed by peribronchovascular interstitium. Lower zone involvement was observed more than upper zone, which was consistent with findings of other studies (2, 22). 

In order to evaluate the severity of lung involvement via CT, we used lobar severity score, which was significantly higher in deceased patients, in comparison with survived group, like Chen et al. and Yuan et al. studies (2, 21). 

An investigation on 121 patients carried out by Bernheim et al. revealed that the longer since the onset of symptoms, CT findings became more prominent (9). Similar to this study, our assessment has demonstrated that patients that presented at earlier stages had lower CTSS. Radiological patterns did not correlate with stage of the disease, which was in contrast to other studies (9, 19). 

The best cut off of chest CT score in predicting mortality in the present study was 12 out of 25, with acceptable sensitivity and specificity of 75.82% and 75.78%, respectively. However, Yang et al. used a different system, scoring 20 pulmonary regions in range of 0-2 with the best cut off point of 19 out of 40 with 94% specificity and 83% sensitivity (2).

Considering shapes of CT scan abnormalities, round opacities correlated with better prognosis. To explain better prognosis of round opacities, more investigation is needed to understand whether this is due to early medical treatment or possible low viral load of the patients as discussed by JSM Peiris et al. for SARS (19, 23). 

Hani et al. demonstrated that CT scan patterns of COVID-19 patients could transform to organizing pneumonia and lung fibrosis as a sequela in advanced phases, which has also been concluded in other studies (24, 25). Therefore, for future follow up of pulmonary artery hypertension secondary to lung injury, we measured MPA, RPA, and LPA diameters as a baseline. Although mean Pulmonary Artery Diameters (PAD) in survived and non-survived group was not beyond the normal range, the differences between them were statically significant. Therefore, it could be used to predict patient prognosis. It is worth noting that the small differences between mean PAD of groups (about 2 millimeters), could raise concerns for operator dependent errors.

Although PAD increase, an indicator of pulmonary hypertension secondary to lung fibrosis, is expected in long term, acute rise of PAD could occur due to lung injury. As mentioned before, patients whose diseases progressed to acute respiratory distress syndrome, showed dilation of pulmonary arteries in days (26).

This research was carried out to explore chest CT scan predictors of prognosis in COVID-19 patients. We found out that radiologic features, especially CT scan severity score, can be helpful in management of patients, along with clinical manifestations. Moreover, we found that pulmonary artery diameter correlated with CT scan severity score and prognosis, although more investigations are needed.

## Limitations

Lack of RT-PCR in patients managed in an out-of-hospital setting, recall bias of patients during phone interviews, interpretation of CT scan images by only one person, differences between Iran's National Guideline for COVID-19 and guidelines of other countries, in addition to usual limitations of cross sectional studies, were among the most important limitations of this study.

## Conclusion:

Based on the results of the present study it seems that there is a significant correlation between chest CT scan characteristics and mortality of COVID-19 cases. Patients with lower CTSS, lower pulmonary artery CT diameter, and round shape opacity had lower mortality. 
